# Who is too fat?

**Published:** 2017

**Authors:** Julien IE Hoffman

## Introduction

Obesity is a modern pandemic that, if not checked, will lead to increased rates of morbidity and mortality from type 2 diabetes, cardiovascular disease, cancer, osteoarthritis, hypertension, and other complications.^1^-^3^ In Tonga, a country with one of the highest percentages of obese people in the world, the recent further increase in incidence of obesity has reduced life expectancy from the mid-70s to the mid-60s.^1^ In the USA, older people with a body mass index (BMI) of 40 kg/m^2^ had an almost four-fold increased mortality rate compared with those with a BMI of 25 kg/m^2^.^4^

A well-executed study by Macia et al.,^5^ published in this edition of the journal, explores some of the factors involved in the obesity pandemic. They examined adults in a rural area in Senegal and compared them with adults in the capital, Dakar. One of the main findings was that there was more overweight and central obesity in the urban than the rural area, and this was attributed to the reduced amount of exercise and higher calorie intake by urban dwellers.

The second important finding was based on showing the subjects silhouettes of people varying from the very obese to the very thin, and asking them what they thought about their weight. Men were more satisfied with their weight than were women. The weight selected as ideal was higher in the rural than the urban area, and for women in the rural area, their perceived ideal weight fell into the overweight category.

In many parts of the world there is improved social status in being fat. This attitude is not confined to underdeveloped countries. In the United Kingdom from 1999 to 2007, the percentage of obese people increased but the proportion who identified themselves as being overweight or obese decreased.^6^

How should we control this pandemic? First, we need to decide who needs treatment. In 1944 Cyril Connolly wrote ‘Imprisoned in every fat man a thin one is wildly signalling to be let out’.^7^ This is a mantra used by many physicians as a reason for lowering weight in all who are overweight. It overlooks, however, the fact that not all fat is created equal. It has long been known that an increase in visceral and abdominal fat (appleshaped) is more deleterious than an increase of subcutaneous fat on the thighs, buttocks and shoulders (pear-shaped),^8^,^9^ and that an increase in visceral fat can ccur with a relatively normal BMI. The two types of fat are functionally different.^10^ It would therefore be more effective to concentrate on treating appleshaped than pear-shaped obesity.

Treatment of obesity follows a simple energy balance: burn up more calories with exercise and take in fewer calories with food. Unfortunately this summation oversimplifies the problem. Changing long-standing habits regarding daily activity and diet is difficult and requires participants to want to change. Furthermore, many obese people have a low metabolic rate that remains low after they have lost weight,^11^ so that a higher proportion of the calories that they eat are stored rather than metabolised. This may be one reason why weight-reducing programmes seldom produce weight loss sustained over many years.

Before we consider recommending weight loss we need to ask ‘Does the subject have the type of obesity that is healthy, and does not need treatment?’ And if it is unhealthy, how do we persuade people who are satisfied with their body shape to change? As physicians, we are quite good at providing care for those who want it, but quite bad at providing care for those who do not want it, such as asymptomatic hypertensives, the mentally ill, and as shown in this article, some of the obese. Treating these groups would do much to decrease morbidity and mortality rates, but we have barely scratched the surface.
